# Cone-Beam Computed Tomography Analysis of Retreatability of Resin and Bio-ceramic Sealer For Root Canal Treatment: An In Vitro Study

**DOI:** 10.7759/cureus.75914

**Published:** 2024-12-17

**Authors:** Ruchali S Bhandare, Sudha B Mattigatti

**Affiliations:** 1 Department of Conservative Dentistry and Endodontics, School of Dental Sciences, Krishna Vishwa Vidyapeeth (Deemed to be University), Karad, IND

**Keywords:** ah plus sealer, angelus bio-c ceramic sealer, calcium silicate-based sealers, cbct, epoxy resin–based sealers, neo-endo and densply retreatment files, re-rct

## Abstract

Root canal retreatment (Re-RCT) cases have shortcomings due to the ineffective removal of the root canal filling material, eventually leading to endodontic failure. This study aims to test the comparative efficacy of retreatment files in removing calcium silicate-based sealer and epoxy resin-based sealer.

Thirty-two single-rooted teeth were decoronated at 15 mm and bio-mechanical preparation was performed. These samples were divided into two groups based on the sealer used - Angelus Bio-C ceramic sealer (Angelus, Londrina-PR, Brazil) and AH Plus sealer (Dentsply Sirona, North Carolina, US). Obturation was done and all the samples were stored in the incubator according to the respective setting time of the sealer. After this, the samples in each group were subdivided into two groups based on the retreatment files used, i.e., Neoendo (Orikam Healthcare India Pvt. Ltd., Haryana, India) and Dentsply (Dentsply Sirona, North Carolina, US) retreatment files. The specimens were then sectioned vertically into two halves and analyzed by cone-beam computed tomography (CBCT).

A one-way ANOVA F test was used for an intragroup comparison, whereas a post hoc Tukey test was used for an intergroup comparison. The Dentsply retreatment files were superior to the Neoendo retreatment files and the Bio-C ceramic sealer was left behind to a greater extent than the AH Plus sealer. Thus, Dentsply retreatment files were more efficient than Neoendo files and the removal of the Bio-C ceramic sealer was more difficult than the AH Plus sealer.

## Introduction

In endodontic root canal failure cases, complete removal of previous root filling material is essential for the success of retreatment. It aims at achieving healthy periapical tissue and relieving the symptoms of the patient [1-3]. The objective is to remove any remnants of the prior obturation process from the root canal system, perform chemical disinfection of the canals, and treat any pathological or iatrogenic deficits [4-6].

Nowadays, a range of sealers are used for obturation. Because of its extensive study in history, AH Plus sealer, which is an epoxy-resin-based endodontic sealer, is regarded as the gold standard and has been used in several comparative studies. Bio-ceramic sealers are becoming more and more common these days. Bio-ceramic sealers are composed of tricalcium silicate (C_3_S), calcium hydroxide [Ca(OH)_2_], zirconium oxide (ZrO_2_), and calcium phosphate monobasic. They exhibit remarkable biocompatibility, good sealing ability, and antimicrobial activity, and also promote periapical tissue mineralization [7].

The capacity of an endodontic sealer to seal is one of its desirable properties. Its easy retrieval from the canal space becomes more important in cases where retreatment is required. There is a wealth of information in the literature about the retrievability of AH Plus sealers. However, there isn't enough information regarding the retrievability of Angelus Bio-C, the more recently available bio-ceramic sealer [8-11].

Various methods have been suggested for removing endodontic materials placed during the root canal treatment. These include using heat, endodontic hand files, sonic and ultrasonic instruments, lasers, Gates Glidden drills, nickel-titanium (NiTi) rotary instruments, and solvents. In the past, removing gutta-percha (GP) by using hand files with or without solvents was a tedious procedure, particularly if the obturating material was inadequately placed. Consequently, the fatigue experienced by the patients and operators during the root canal retreatment may be reduced by using NiTi rotary devices [12].

The effectiveness of retreatment can be evaluated by sectioning the teeth and then examining the results under a stereomicroscope, dental operating microscope, or scanning electron microscope. However, these methods require invasive procedures. They can also be inaccurate because there is a chance some of the leftover material will be lost if the teeth splits up [13]. Cone-beam computed tomography (CBCT) has been suggested recently as a noninvasive way for evaluating retreatment procedures quantitatively [14]. It enables a precise assessment of root filling removal without causing any harm to the tooth. Studies utilizing CBCT to evaluate retreatment with bio-ceramic sealers are currently rare.

## Materials and methods

Specimen selection

**Figure 1:**
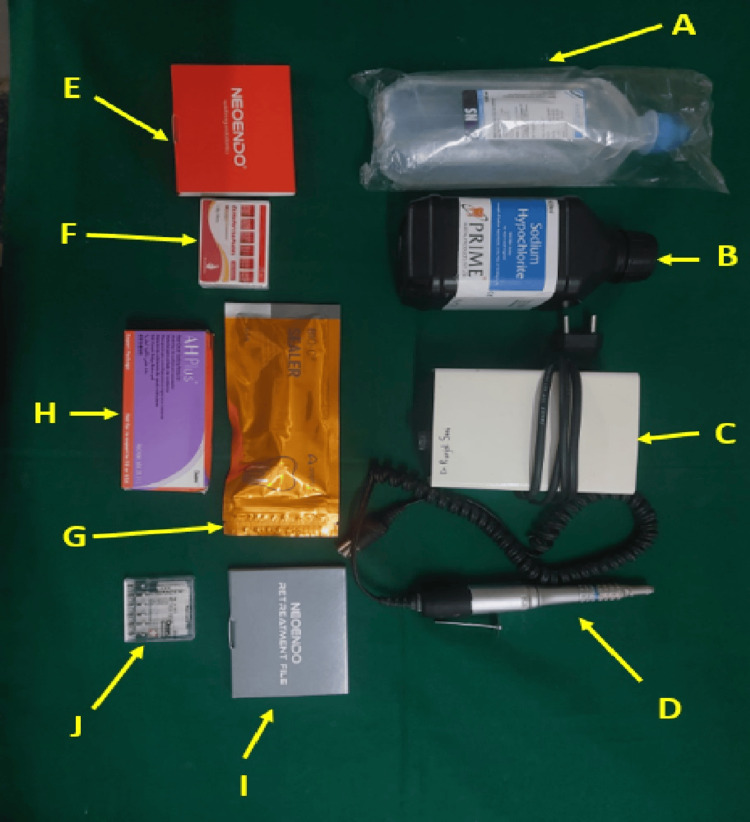
Materials and methods used for the retreatment A. Normal saline B. Sodium hypochlorite (Prime Dental Products Pvt. Ltd., Maharashtra, India) C. Micromotor (Marathon, Saeyang, Korea) D. Straight handpiece (Marathon, Saeyang, Korea) E. Neoendo rotary files (Orikam Healthcare India Pvt. Ltd., Haryana, India) F. Gutta-percha (Sure endo, Korea) G. Bio-ceramic sealer (Bio-C, Angelus, Londrina-PR, Brazil) H. AH Plus sealer (Dentsply Sirona, North Carolina, US) I. Neoendo retreatment files (Orikam Healthcare India Pvt. Ltd., Haryana, India) J. Dentsply retreatment files (Dentsply Sirona, North Carolina, US)

From a collection of extracted teeth, 32 single-rooted teeth were chosen. The inclusion criteria comprised teeth extracted for orthodontic treatment, periodontal disease, and enamel and dentin carious teeth. Teeth with severe decay or root caries and those with fractured or curved roots were excluded. The soft tissue remnants and caries on the root surface were mechanically removed. The materials used for the study are shown in Figure 1. 

**Figure 2:**
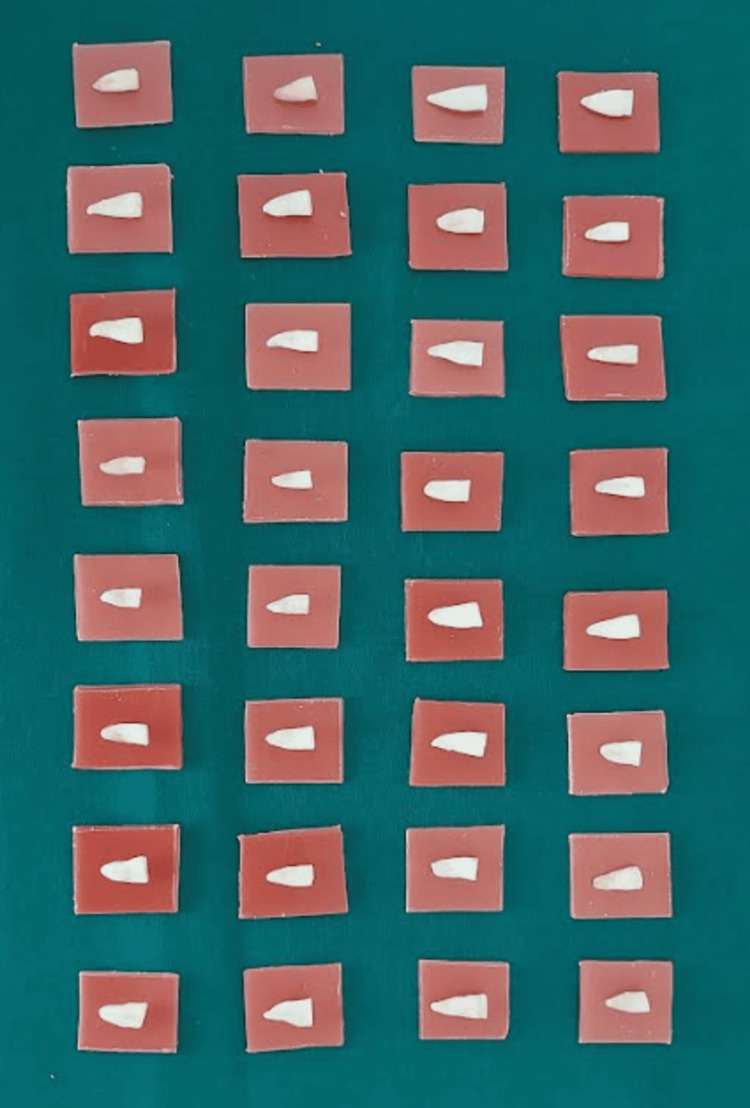
Tooth samples

The tooth samples included in the testing are shown in Figure 2.

Initial endodontic treatment

Standardization of the root length (15 mm) was done with the help of a low-speed diamond disc. The working length (WL) of the canal was recorded by deducting 1 mm from the root length, i.e., 14 mm. To confirm the existence of a single canal, radiographs of the teeth were taken at various angulations. Neoendo rotary instruments up to a size, 25/0.06 taper, were used for the root canal procedures. All the canals were irrigated with saline and 5.25% sodium hypochlorite after each step throughout the instrumentation process. Following chemo-mechanical procedure, 17% ethylenediaminetetraacetic acid (EDTA) and 5.25% sodium hypochlorite were used for the removal of the smear layer. Paper points were used to dry the canals. Obturation was done using single cone obturation technique with GP and sealer. After that, the teeth were kept in an incubator at 37°C for eight hours and two hours in the case of the AH Plus and Angelus Bio-C sealers, respectively, for them to set completely [10]. 

Treatment groups

After the sealers were completely set, the teeth samples were split into two groups of 16 teeth each:

Group A - They were obturated using a single GP cone and AH Plus sealer.

Group B - They were obturated using a single GP cone and Bio-C ceramic sealer.

Each group was then separated into two subgroups of eight teeth each, based on the retreatment files used as follows:

Subgroup 1 - Dentsply retreatment file system

Subgroup 2 - Neoendo retreatment file system

Retreatment procedure

The obturation material was removed using Dentsply retreatment files D1, D2, D3, and Neoendo retreatment files N1, N2, N3. The retreatment file was forwarded until the working length was reached.

Cone-beam computed tomography (CBCT) analysis

To evaluate the remnants, CBCT scans were performed both before and after the retreatment. The proportion of the residual filling material in the coronal-third, middle-third, and apical-third of the root canals were scored using the grading system given by Somma et al. [15].

1. Score 1: 0-25% means slight debris or no debris on the dentin surface

2. Score 2: 25-50% means debris present on the dentin surface

3. Score 3: 50-75% means moderate amount of debris present on the dentin surface

4. Score 4: >75% means heavy amount of debris present on the dentin surface

## Results

We aimed to compare the efficacy of the retreatment files with the help of CBCT which revealed the remnant material present in the canal. Intragroup comparison was done by one-way ANOVA F test and intergroup comparison was done by post hoc Tukey test. An unpaired t-test (also known as an independent t-test) is a statistical procedure that compares the averages/means of two independent or unrelated groups to determine if there is a significant difference between the two.

Table 1 shows the remnant material of AH Plus sealer by using Dentsply and Neoendo retreatment files after CBCT analysis. It revealed that Dentsply retreatment files were superior to Neoendo files in removing the AH Plus sealer.

**Table 1:** Statistical comparison of the volumetric CBCT results of remaining material (%) using AH Plus sealer with Dentsply retreatment file vs Neoendo retreatment file system CBCT: cone-beam computed tomography; Unpaired t-test: p>0.05, not significant; *p<0.05, significant; **p<0.001, highly significant.

Group A (AH Plus)	Mean	Standard deviation	Unpaired t-test	P value, significance
Group A1 (AH Plus+Dentsply)	3.01	0.27	t=-12.896	p<0.001**
Group A2 (AH Plus+Neoendo)	5.02	0.34		

Table 2 shows the remnant material of Angelus Bio-C sealer by using Dentsply and Neoendo retreatment files after CBCT analysis. It revealed that Dentsply retreatment files were superior to Neoendo files in removing the Angelus Bio-C sealer.

**Table 2:** Statistical comparison of volumetric CBCT results of remaining material (%) using Angelus Bio-C sealer with Dentsply retreatment file vs Neoendo retreatment file system CBCT: cone-beam computed tomography; Unpaired t-test: p>0.05, not significant; *p<0.05, significant; **p<0.001, highly significant.

Group B (Angelus Bio-C)	Mean	Standard deviation	Unpaired t-test	P value, significance
Group B1 (Angelus Bio-C+Dentsply)	7.23	0.65	t=-10.838	p<0.001**
Group B2 (Angelus Bio-C+Neoendo)	10.03	0.32		

The comparative efficacy of Dentsply retreatment files in the removal of AH Plus sealer and Angelus Bio-C sealer using CBCT (Table 3) revealed that it was difficult to remove the Angelus Bio-C sealer as compared to the AH Plus sealer.

**Table 3:** Statistical comparison of volumetric CBCT results of the remaining material (%) with the two sealers using the Dentsply file system CBCT: cone-beam computed tomography; Unpaired t-test: p>0.05, not significant; *p<0.05, significant; **p<0.001, highly significant.

Subgroup 1 (Dentsply file system)	Mean	Standard deviation	Unpaired t-test	P value, significance
Group A1 (AH Plus+Dentsply)	3.01	0.27	t=-16.786	p<0.001**
Group B1 (Angelus Bio-C+Dentsply)	7.23	0.65		

The comparative efficacy of Neoendo retreatment files in the removal of AH Plus sealer and Angelus Bio-C sealer using CBCT (Table 4) revealed that it was difficult to remove the Angelus Bio-C sealer as compared to AH Plus sealer. 

**Table 4:** Statistical comparison of volumetric CBCT results of the remaining material (%) with the two sealers using the Neoendo file system CBCT: cone-beam computed tomography; Unpaired t-test: p>0.05, not significant; *p<0.05, significant; **p<0.001, highly significant.

Subgroup 2 (Neoendo file system)	Mean	Standard deviation	Unpaired t-test	P value, significance
Group A2 (AH Plus+Neoendo)	5.02	0.34	t=-30.092	p<0.001**
Group B2 (Angelus Bio-C+Neoendo)	10.03	0.32		

**Figure 3:**
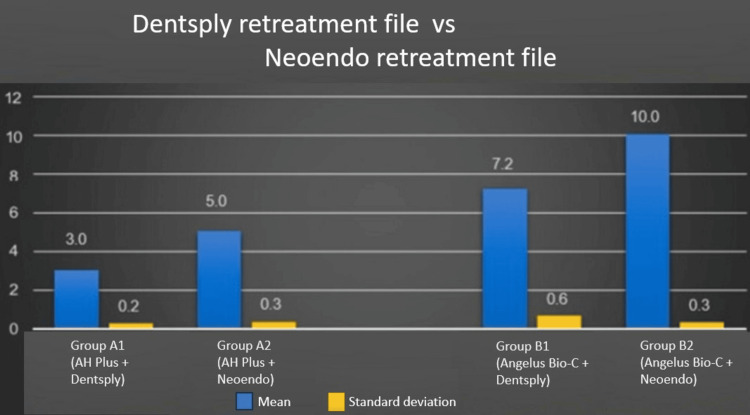
Dentsply retreatment files vs Neoendo retreatment files

The same results can be seen in the graphs. Figure 3 depicts the residual filling material with the Dentsply retreatment files and the Neoendo retreatment files when the AH Plus sealer and Angelus Bio-C sealers were used. The Neoendo retreatment files were inferior in removing the filling material compared to the Dentsply retreatment files and the removal of the AH Plus sealer was easier than the Angelus Bio-C sealer.

**Figure 4:**
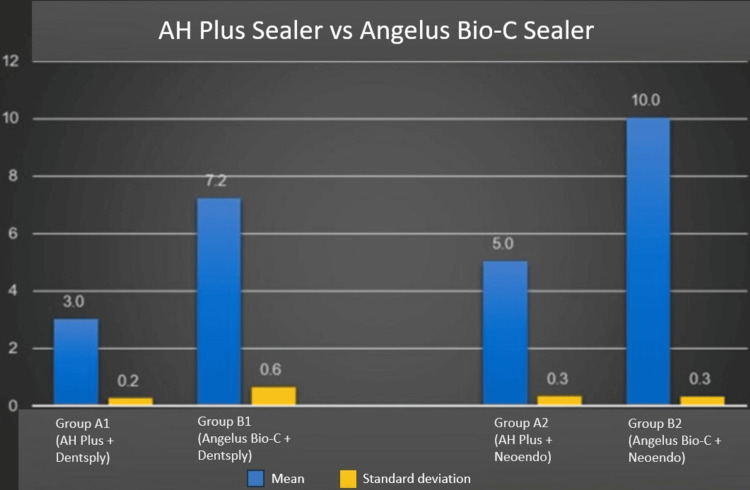
AH Plus sealer vs Angelus Bio-C sealer

Figure 4 shows that the AH Plus sealer can be easily removed with Dentsply retreatment files as compared to the other subgroups.

**Figure 5:**
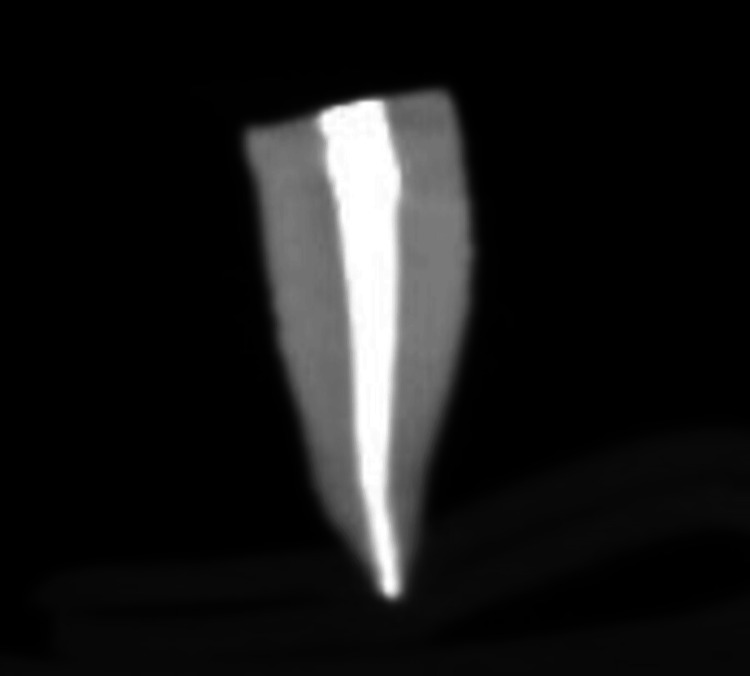
CBCT image after obturating with AH Plus sealer The white continuous shade (radio-opacity) at the center of the tooth shows the obturating material (gutta-percha along with AH Plus sealer). CBCT: cone-beam computed tomography

**Figure 6:**
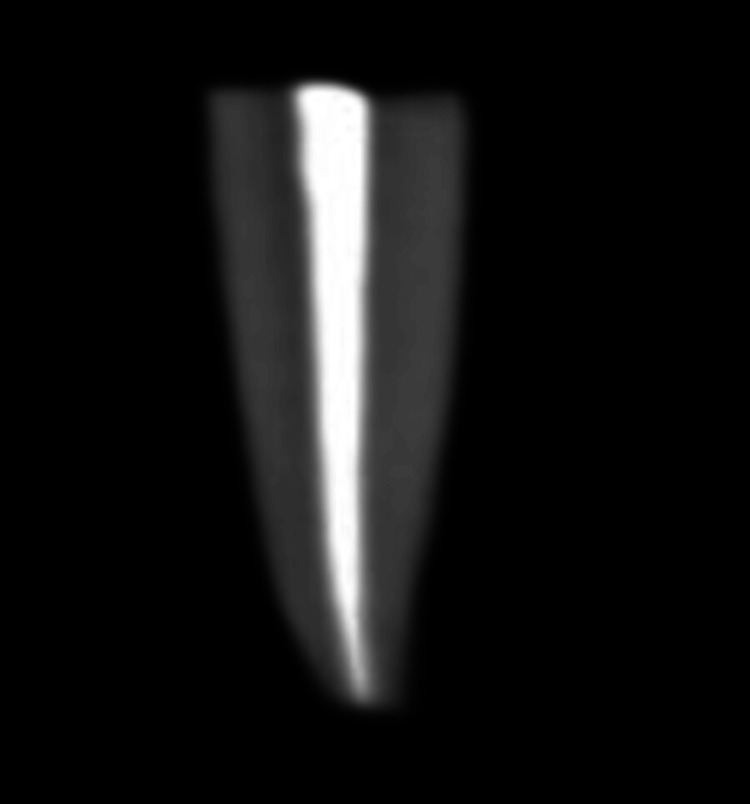
CBCT image after obturating with the Angelus Bio-C sealer The white continuous shade (radio-opacity) at the center of the tooth shows the obturating material (gutta-percha along with the Angelus Bio-C sealer). CBCT: cone-beam computed tomography

CBCT was used to check the proper sealing of the obturating materials. The figures below show the obturation using the AH Plus sealer (Figure 5) and the Angelus Bio-C sealer (Figure 6) with GP.

**Figure 7:**
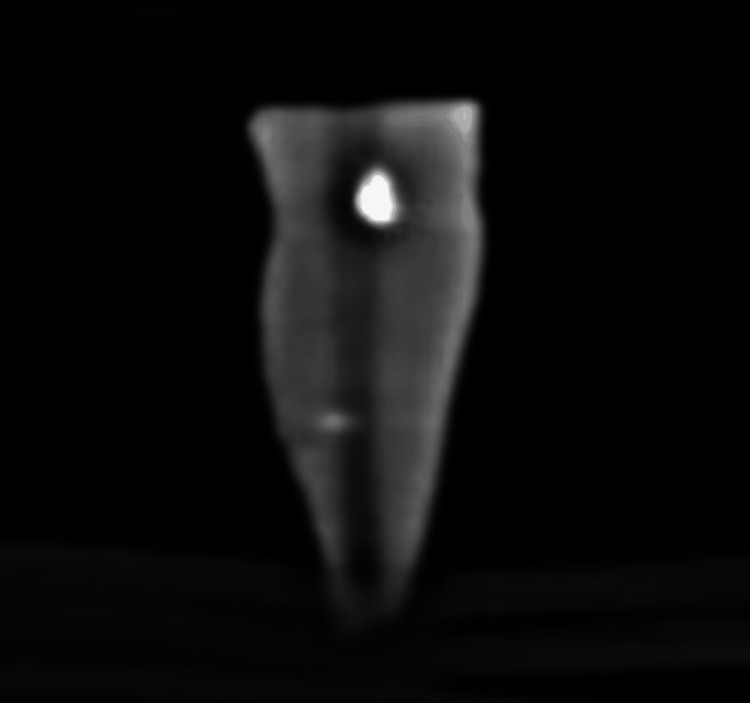
CBCT image after using the Dentsply retreatment files and obturating with AH Plus sealer The white discontinuous shade (radio-opacity) at the center of tooth shows the remnant of the AH Plus sealer after retreatment with the Dentsply files. CBCT: cone-beam computed tomography

Figure 7 shows the remnant of AH Plus sealer after the use of the Dentsply retreatment files.

**Figure 8:**
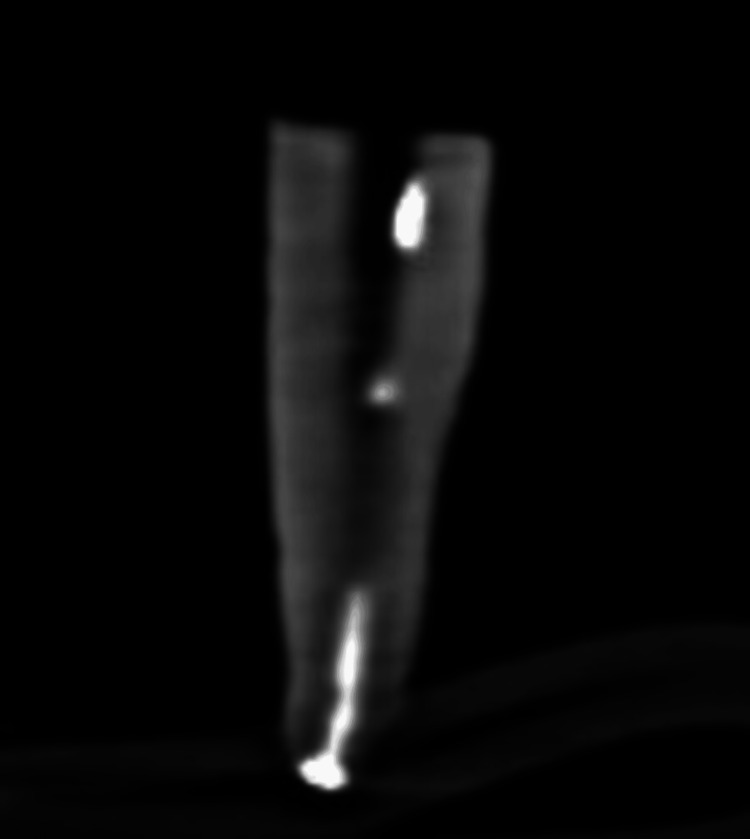
CBCT image after using the Dentsply retreatment files and obturating with the Angelus Bio-C sealer The white discontinuous shade (radio-opacity) at the center of tooth shows the remnant of the Angelus Bio-C sealer after retreatment with the Dentsply files. CBCT: cone-beam computed tomography

Figure 8 shows the remnant of the Angelus Bio-C sealer after the use of the Dentsply retreatment files.

**Figure 9:**
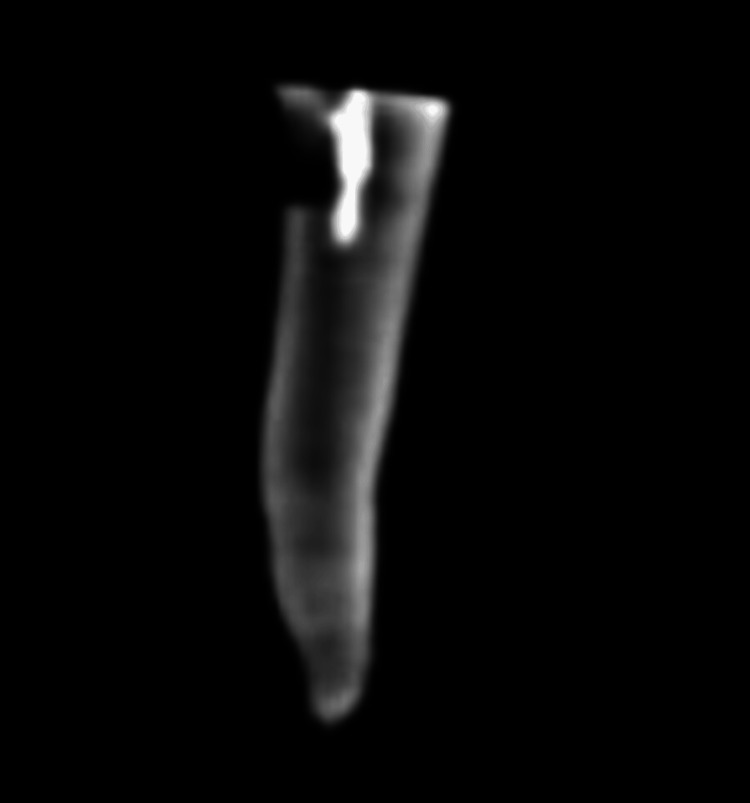
CBCT image after using the Neoendo retreatment files and obturating with the AH Plus sealer The white discontinuous shade (radio-opacity) at the center of the tooth shows the remnant of the AH Plus sealer after retreatment with the Neoendo files. CBCT: cone-beam computed tomography

Figure 9 shows the remnant of the AH Plus sealer after the use of the Neoendo retreatment files.

**Figure 10:**
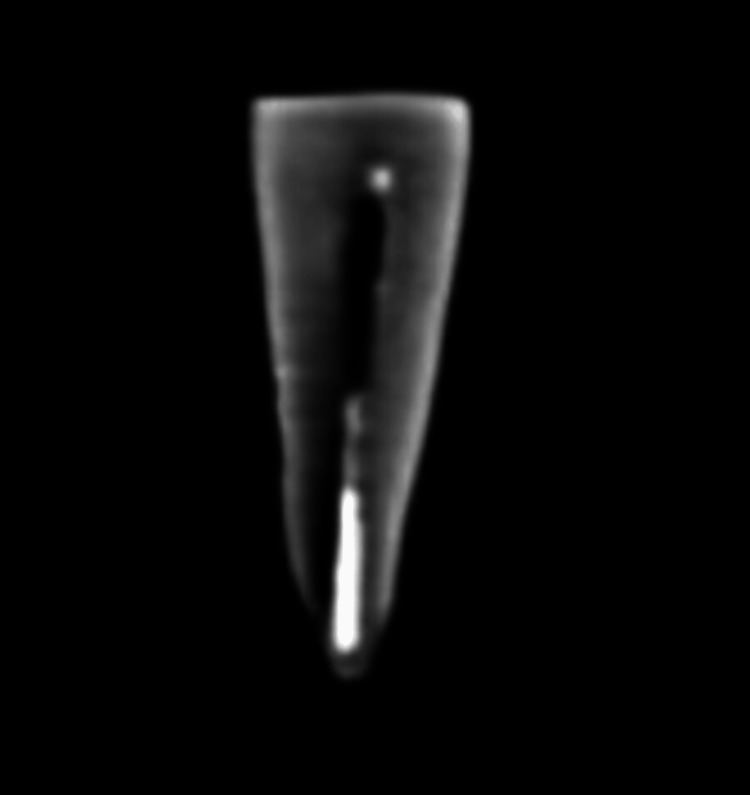
CBCT image after using the Neoendo retreatment files and obturating with the Angelus Bio-C sealer The white discontinuous shade (radio-opacity) at the center of the tooth shows the remnant of the Angelus Bio-C sealer after retreatment with the Neoendo files. CBCT: cone-beam computed tomography

Figure 10 shows the remnant of the Angelus Bio-C sealer after the use of the Neoendo retreatment files.

## Discussion

The prerequisites for successful nonsurgical root canal retreatment (re-RCT) are the removal of complete root canal material, improved debridement, and three-dimensional obturation [16]. The majority of research examining the removal of different root-filling materials states that it is impossible to remove the entire material. However, in order to effectively perform retreatment, the working length and apical patency need to be restored [17].

It has been discovered that the use of rotary instruments to remove root-filling materials during retreatment is safer and faster, resulting in shorter clinical time and lesser operator fatigue [18]. It has been demonstrated that they are more effective than manual techniques in creating a tapered root canal, with the least amount of abnormalities and canal transportation. Dentsply retreatment files were used in this study. Studies assessing the effectiveness of the Dentsply retreatment files are fewer. It is said that the Dentsply retreatment files are quite effective in removing the sealer and GP during retreatment. This result may be explained by the superior sharpness of these files. Dentsply files are available in three types: D1, D2, and D3. D1 has a cutting tip to help with the initial preparation, while D2 and D3 have non-cutting tips, which remove root-filling material from the middle-third and apical-third portions. They have convex triangular cross sections. The GP is drawn into the flutes of the file and directed toward the canal opening, which facilitates more effective cutting. The Neoendo retreatment files, on the other hand, have positive rake angles and parallelogram cross sections. Due to this, the contact between the dentin and the file is restricted to one or two points at the cross section [19-22].

Prior research suggests that sectioning teeth in the longitudinal direction was required for the retreatment study of the remaining filling material, which was then accompanied by scanning electron microscopy examination or digital surface imaging. These techniques, however, are two-dimensional and do not provide a precise way to evaluate the amount of root filling left within the root canals. In endodontics, micro-CT has been recently employed as a research tool to examine root canal anatomy, evaluate root canal preparation methods, evaluate the effectiveness of obturating techniques, and remove root-filling materials [23-25]. Micro-CT, however, is not appropriate for clinical application. Therefore, it is critical to assess the techniques of root-filling material removal to select a method that demonstrates accuracy comparable to micro-CT while being therapeutically practical. According to a recent study, as a clinical substitute for micro-CT, CBCT tools with excellent resolution are the only ones that enable dentists to identify the root canal length [26]. Furthermore, it has been demonstrated that spiral CT and peripheral quantitative CT are less precise than CBCT scans [27].

Nowadays, root canal obturation is increasingly done with calcium silicate sealers. The tricalcium silicate (C_3_S)-based Angelus Bio-C sealer, available in the market, is composed of a liquid containing calcium chloride and polycarboxylate polymer, and a fine powder of tricalcium silicate and zirconium oxide [28,29]. It is well known that calcium silicate sealers harden after setting. They may be more adherent and resistant to dislocation from the dentin. Their unique setting activity is due to the hydration reaction that occurs when it comes in contact with water, which leads to precipitation reaction of calcium phosphate, and prevents them from being removed during retreatment. Therefore, it is clinically relevant to assess the retreatability of Angelus Bio-C sealers [30].

There have been reports of similarities in dentinal adhesion between AH Plus and Angelus Bio-C sealers. But, in the case of Angelus Bio-C sealer, apatite and cement plugs precipitate within the dentinal tubules, creating a mineral infiltration zone of up to 2000 µm. This results in a micromechanical and chemical attachment that prevents the sealer from dislocating from the dentin. On the other hand, the AH Plus sealer penetrates into the dentinal tubules but lacks the mineral infiltration zone [31].

## Conclusions

Successful endodontic retreatment relies on the effective removal of the existing obturation material, which is essential for restoring access to the apical foramen, achieving optimal disinfection, and promoting the healing of periapical tissues. Advances in instrumentation, such as the NiTi rotary systems, have greatly improved retreatment outcomes by reducing clinical time and operator fatigue compared to the traditional hand files. Additionally, various sealers, such as AH Plus and the newer bio-ceramic formulations like Angelus Bio-C sealer, present unique properties that influence retrievability and biocompatibility. While the AH Plus sealer remains a well-established standard, bio-ceramic sealers offer promising benefits in terms of biocompatibility and periapical healing, though their resistance to removal during retreatment raises considerations for future clinical research.

Furthermore, the development of noninvasive imaging modalities like CBCT has provided clinicians with improved capabilities to assess the effectiveness of retreatment without compromising the integrity of the tooth. This is especially relevant as it overcomes the limitations of traditional two-dimensional assessments in accurately determining the extent of the residual filling materials. Studies indicate that CBCT may offer a clinically practical alternative to micro-CT in evaluating root filling removal, thereby enhancing retreatment precision. As bio-ceramic sealers and advanced imaging technologies evolve, additional research is essential to optimize their application, providing more predictable retreatment outcomes and enhancing patient care in endodontics.
